# Association of intraoperative hyperglycemia with postoperative composite infection after cardiac surgery with cardiopulmonary bypass: A retrospective cohort study

**DOI:** 10.3389/fcvm.2022.1060283

**Published:** 2023-01-13

**Authors:** Xinglong Xiong, Dongxu Chen, Shuang Cai, Li Qiu, Jing Shi

**Affiliations:** ^1^Department of Anesthesiology, The Affiliated Hospital of Guizhou Medical University, Guiyang, China; ^2^Department of Anesthesiology, West China Hospital, Sichuan University, Chengdu, China

**Keywords:** glucose, infection, cardiac surgery, cardiopulmonary bypass, hyperglycemia

## Abstract

**Background:**

The association between intraoperative hyperglycemia (IH) and postoperative infections in patients undergoing cardiac surgery with cardiopulmonary bypass (CPB) is inadequately studied.

**Methods:**

A total of 3,428 patients who underwent cardiac surgery with CPB at our institution between June 1, 2019 and July 30, 2021 were enrolled to evaluate the association of IH (blood glucose ≥ 180 mg/dL) with postoperative infection in patients. The new onset of any type of infection and the optimal cutoff values of intraoperative glucose to predict in-hospital infection were determined.

**Results:**

The composite outcome occurred in 497 of 3,428 (14.50%) patients. IH was associated with an increased risk of postoperative composite infection [adjusted odds ratio: 1.39, (95% confidence interval), 1.06–1.82, *P* = 0.016]. Restricted cubic splines were applied to flexibly model and visualize the association of intraoperative peak glucose with infection, and a J-shaped association was revealed. Besides, it was demonstrated that the possibility of infection was relatively flat till 150 mg/dL glucose levels which started to rapidly increase afterward.

**Conclusion:**

We summarize that IH is associated with an elevated risk of postoperative new-onset composite infections and perioperative blood glucose management should be more stringent, i.e., lesser than 150 mg/dL in patients undergoing cardiac surgery.

## 1. Introduction

Infection after cardiac surgery is common and potentially fatal (1). The incidence of infection affects 10% of patients after cardiac surgery, such as pulmonary, blood, and urinary (2). Postoperative infections can impair functional recovery, delay postoperative ambulation, and increase the risk of mortality (3). Studies have stated that postoperative infections increase postoperative mortality from 3.5% to 16.8%, notably, with an almost 9-fold increase in mortality of patients with pulmonary infection (3, 4). Therefore, it is important to identify potentially modifiable factors that may result in postoperative infection after cardiac surgery.

Of late, the management of perioperative hyperglycemia has gained much interest (5). Intraoperative hyperglycemia (IH) following cardiac surgery is common; hence, recent years have intensively focused on glycemic control during all cardiac surgical procedures (6, 7). Moreover, several studies have demonstrated that glucose control could reduce morbidity and improve patient outcomes (8). However, there has been a continuous debate to recommend the target value of glucose control (9). Although the negative effects of hyperglycemia in patients following coronary artery bypass graft (CABG) have been established, the optimal value of glucose control has not yet been clearly elucidated and remains inconclusive (10). Besides, the guidelines of the Society of Thoracic Surgeons (STS) recommend that diabetic and non-diabetic patients undergoing cardiac surgery should maintain intraoperative blood glucose concentration (BGC) of less than 180 mg/dL (11), suggesting that tight glycemic control could improve outcome. However, the subsequent landmark NICE-SUGAR study revealed that tight glucose control (81-108 mg/dL) resulted in a higher risk of mortality than moderate glucose control (140-180 mg/dL) in patients in the medical-surgical intensive care unit (12). As the largest randomized control trial in this field to date, the NICE-SUGAR study offered strong evidence against tight glucose control in critically ill patients, while methodological flaws in the protocol in terms of insulin administration contributed to a higher risk of hypoglycemia was observed (13). Therefore, there has been a great interest in discerning optimal serum glucose levels. But the optimal value of glucose control has not yet been clearly elucidated and the effect of IH after cardiac surgical procedures on all types of infectious complications is inadequately studied. The paucity of such data hinders an evidence-based approach to improving intraoperative glucose management.

In this retrospective observational cohort study, we assessed the association of IH after cardiac surgery with cardiopulmonary bypass (CPB) with the occurrence of composite infections. We hypothesized that the presence of IH (defined as BGC ≥ 180 mg/dL) was independently associated with postoperative infection. Furthermore, the study also evaluated optimal cutoff values of IH to predict infections.

## 2. Materials and methods

### 2.1. Study design and participants

Medical data were retrospectively collected for patients who had been admitted to West China Hospital of Sichuan University (Chengdu, China). All variables retrieved from the Hospital Electronic Medical Record System and medical records. The quality of the study was ensured by following the guidelines of Strengthening the Reporting of Observational Studies in Epidemiology (STROBE) (14). Moreover, the institutional review boards at West China Hospital of Sichuan University (No. 869/2021, July 23, 2021) approved the study and a waiver of informed consent. The analysis plan was written after the data had been accessed. The analysis plan was written after the data had been accessed. The study was registered at the Chinese Clinical Trial Registry (ChiCTR2100049358, date of registration: August 1, 2021).

**FIGURE 1:**
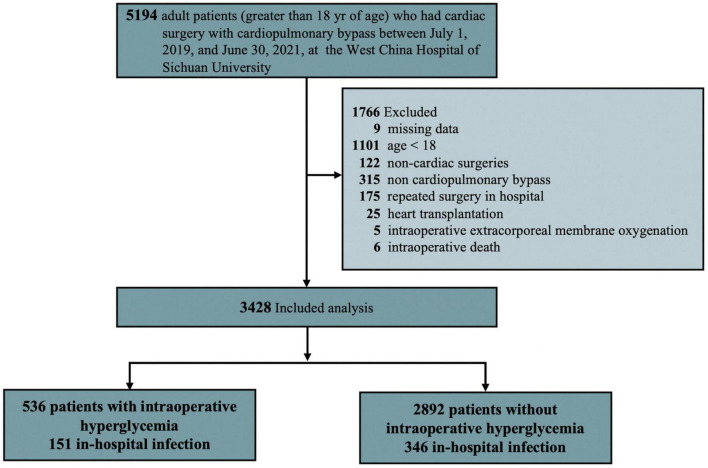
Flow chart of patient enrollment.

Data for adult patients (greater than 18 years) who had cardiac surgery with CPB between June 1, 2019 and July 30, 2021 were included in the study. However, the patients who had heart transplantation, > 4 missing intraoperative blood glucose data, did not survive the operation, received an intra-aortic balloon pump or extracorporeal membrane oxygenation for weaning off CPB in the operating room, and had invalid, incomplete, or unavailable outcome were excluded from the study (Figure 1).

### 2.2. Anesthesia and CPB management

The common and standard procedures of anesthesia and CPB were applied as described (15). In brief, anesthesia was induced with midazolam, sufentanil, muscle relaxant, and/or etomidate, and maintained by continuous remifentanil infusion and sevoflurane inhalation on intravenous propofol, combined with intermittent muscle relaxant and sufentanil intravenous injection. In addition, central venous pressure, arterial catheter, SpO_2_, end-tidal CO_2_, and arterial blood gas were monitored throughout the operation.

CPB involved a roll pump (Sarns 8000, 3M; Soma Technology Inc., Bloomfield, CT), a membrane oxygenator (Medtronic Inc., Minneapolis, MN), a filter (Dongguan Kewei Medical Instrument Co., Ltd., Dongguan, China) and connecting tubes (Tianjin Plastic Research Institute, Tianjin, China), and was primed with 4% succinylated gelatin solution of 1,000 mL and 500 mL crystalline liquid. Next, to maintain mean arterial pressure greater than 50 mmHg during CPB, blood flow was set as 2.0-2.4 L/m^2^/min. Meanwhile, cold 4:1 blood cardioplegia was applied for heart arrest. Besides, the nasopharyngeal temperature was maintained with a moderate hemodilution strategy at 32–34°C. Subsequently, all patients received an initial heparin dose of 375 U/kg to achieve systemic anticoagulation. Moreover, to maintain activated clotting time longer than 480 s, additional heparin was injected intermittently. Eventually, protamine in a 1:1 ratio neutralized heparin based on the initial heparin dose while weaning from CPB (15). Moreover, red blood cells were transfused in the following cases; hemoglobin lower than 7 g/dL during CPB, 8 g/dL during operation without CPB, or the attending physician ordered transfusion based on the patient’s clinical condition (16). Other blood products would be administered based on the presence of ongoing bleeding or abnormal coagulation occurred. During the surgical procedure, pericardial blood was salvaged by suction and returned to the CPB circuit. Subsequently, residual blood was collected in a bag containing sodium citrate, neutralized by protamine, and transfused into the patient after the termination of CPB.

### 2.3. Intraoperative blood glucose analysis

In current study, preoperative glycemic management was Chinese Expert Consensus on Perioperative Blood Glucose Management (2016), it was consistent with the ESC, AHA guideline (17, 18). We recorded all glucose values during the procedure. BGCs were determined by arterial blood glucose analysis (mg/dL, tested by Roche Cobas b 123 blood gas and electrolytes analyzer). We defined IH as at least occurred one arterial sample with a BGC ≥ 180 mg/dL (11). The prevalence of IH was calculated by the number of hyperglycemic patients divided by the total number of patients enrolled. The management of IH to initiate insulin therapy was determined at the discretion of the anesthesia teams of the patients.

### 2.4. Variables and outcome

Investigators collected data from the institutional databases using a standardized data collection form. The data collected included patient-specific variables, surgery-specific variables, preoperative medications, and laboratory findings. Additionally, Age-adjusted Charlson Comorbidity Index (ACCI) was calculated, as a proxy of baseline somatic fitness, summarizing the presence of 18 medical conditions comorbidities were identified from Electronic Health Records (19), and European System for Cardiac Operative Risk Evaluation II (EuroSCORE II) was also evaluated (20).

The outcome was the new onset of composite in-hospital infection, which included lung, wound, blood, and urinary tract infection in hospital after cardiac surgery with CPB. The new onset of composite in-hospital infection after surgery was determined by medical record review. Composite infection was defined as a combination of all types of newly diagnosed infection after surgery. If more than one complication occurred after surgery, only the initial complication was included in the analysis of composite infection. The diagnosis of specific infections was determined in accordance with the definitions and criteria established by the European Perioperative Clinical Outcome (EPCO) definitions (details shown in Appendix Table A in Supplementary material) (21). Outcome diagnosis was strictly determined according to pre-established definitions. All data were inspected to guarantee reliability, and data collocation procedures were performed by two independent investigators.

## 3. Statistical analysis

Continuous variables were reported as mean + standard deviation (SD) or as the median and interquartile range (IQR), while categorical variables were expressed as number and percentage. Intergroup differences in continuous variables were assessed for significance using Student’s *t* test, or the Wilcoxon rank-sum test if the data were skewed. Qualitative data were compared using a chi-squared test or Fisher’s exact test, as appropriate.

The association between the IH and the risk of any infection was assessed using odds ratios (ORs) with 95% confidential intervals (CIs), derived from logistic regression models. Models were partly (models 1-4) or fully (model 5) adjusted for demographic factors (age, sex, and body mass index [BMI]), preoperative comorbidities (i.e., ACCI, EuroSCORE II, diabetes mellitus, congenital heart disease, chronic obstructive pulmonary disease, etc.), operative data (i.e., operation type, anesthesia method, operation duration, etc.), preoperative laboratory test (i.e., hemoglobin, N-terminal pro-brain natriuretic peptide, Cardiac troponin T, etc.), intraoperative fluid management and vasoactive agents (i.e., epinephrine, milrinone, intraoperative red blood cell infusion, etc.) (see details in Table 1 and Appendix Table B in Supplementary material). Subgroup analysis was also performed by age group, sex, BMI, ACCI, EuroSCORE II, New York Heart Association (NYHA) class, preoperative glucose, operation duration, type of surgery and anesthesia method.

**TABLE 1:** Demographics and clinical characteristics stratified by composite primary outcome.

Demographics	All *N* = 3428	Any infection		*P*-value
		**Yes (*n* = 497)**	**No (*n* = 2931)**	
**Demographic characteristics**				
Age, years, median [IQR]	53.00 [46.00; 61.00]	55.00 [48.00; 64.00]	53.00 [45.00; 61.00]	<0.001^a^
Sex, *n* (%)				<0.001^b^
Male	1,700 (49.59)	298 (59.96)	1402 (47.83)	
Female	1,728 (50.41)	199 (40.04)	1529 (52.17)	
BMI, kg/m^2^, median [IQR]	23.24 [21.09; 25.46]	23.67 [21.36; 25.95]	23.19 [21.05; 25.39]	0.030^a^
**Preoperative comorbidities**				
Aged-adjusted Charlson, mean (SD)	1.493 (1.34)	1.805 (1.42)	1.440 (1.32)	<0.001^c^
EuroSCORE II, mean (SD)	2.718 (2.80)	4.231 (3.32)	2.461 (2.62)	<0.001^c^
ASA, *n* (%)				<0.001^b^
< III	3,136 (91.48)	377 (75.86)	2759 (94.13)	
> III	29 (8.52)	120 (24.14)	172 (5.87)	
NYHA, *n* (%)				<0.001^b^
1	262 (7.64)	17 (3.41)	245 (8.36)	
2	1,865 (54.40)	239 (48.09)	1626 (55.48)	
3	1,208 (35.24)	212 (42.66)	996 (33.98)	
4	93 (2.72)	29 (5.84)	64 (2.18)	
CHD, *n* (%)	288 (8.40)	60 (12.07)	228 (7.78)	0.002^b^
DM, *n* (%)	204 (5.95)	42 (8.45)	162 (5.53)	0.014^b^
COPD, *n* (%)	148 (4.32)	33 (6.64)	115 (3.92)	0.008^b^
Unstable angina, *n* (%)	71 (2.07)	19 (3.82)	52 (1.77)	0.005^b^
Recent myocardial infarct^†^, *n* (%)	61 (1.78)	16 (3.22)	45 (1.54)	0.015^b^
Pulmonary hypertension^‡^, *n* (%)	216 (6.30)	52 (10.46)	164 (5.56)	<0.001^b^
Previous cardiac surgery, *n* (%)	253 (7.38)	64 (12.88)	189 (6.45)	<0.001^b^
**Operative data**				
Emergency, *n* (%)	255 (7.44)	94 (18.91)	161 (5.49)	<0.001^b^
Operation duration, min, median [IQR]	245.00 [205.00; 305.00]	319.00 [240.00; 414.00]	240.00 [200.00; 291.00]	<0.001^a^
CPB duration, min, median [IQR]	121.00 [90.00; 162.25]	163.00 [118.00; 220.00]	116.00 [88.00; 152.00]	<0.001^a^
Operation type*, *n* (%)				<0.001^b^
CABG	215 (6.27)	170 (5.80)	45 (9.05)	
Isolated valve	1,065 (31.07)	941 (32.11)	124 (24.95)	
Multi valve	194 (5.66)	163 (5.56)	31 (6.24)	
Vascular	513 (14.96)	378 (12.90)	135 (27.16)	
CHD	267 (7.79)	253 (8.63)	14 (2.82)	
Mixed	967 (28.21)	831 (28.35)	136 (27.36)	
Others	207 (6.04)	195 (6.65)	12 (2.42)	
Intraoperative hyperglycemia, *n* (%)	536 (15.64)	151 (30.38)	385 (13.14)	<0.001^b^
The peak glucose, mg/dl, mean (SD)	139.40 (43.53)	158.27 (52.42)	136.21 (40.99)	<0.001^c^

Data are present in *n* (%), mean (SD) or median [IQR]. ^†^Recent myocardial infarction is defined as any diagnosed myocardial infarction in past 90 days before the operation. ^‡^Pulmonary hypertension is defined as pulmonary artery systolic pressure equal or more than 31 mmHg. *For operation type: mixed operations are referred to operations combined with CABG, and/or valve, and/or CHD, and/or others; others are referred to operations that could not be included in the types above (i.e., cardiac myxoma, pulmonary endarterectomy, MAZE, etc.) *P*-values are derived from: ^a^ u-test, ^b^ chi-square test, ^c^ *t*-test. ASA, American Society of Anesthesiologists classification; BMI, body-mass index; CABG, coronary artery bypass grafting; CHD, congenital heart disease; COPD, chronic obstructive pulmonary disease; CPB, cardiopulmonary bypass; DM, diabetes mellitus; EuroSCORE II, European system for cardiac operative risk evaluation II; IQR, interquartile range; NYHA, New York Heart Association Classification.

The multivariable relationship between the peak glucose during surgery and infection was assessed using adjusted OR curves. Linearity between the peak glucose during surgery and response was modeled by a restricted cubic spline (RCS) function with three knots located at 10th, 50th, and 90th percentiles, and with fully covariates adjusted (model 5). The level of the peak glucose during surgery that corresponded to the OR value of 1.00 was defined as the change point of the infection incidence. We also performed subgroup analysis by baseline glucose (≤ 126 or > 126 mg/dL, i.e., 7.0 mmol/dL) (22) and history of diabetes mellitus (yes or no). The differences in ORs were assessed by introducing an interaction term to the logistic models or by the Wald test. During the peer-review process, reviewers suggested that a subgroup analysis should be performed by age group, sex, BMI, ACCI, EuroSCORE II, NYHA class, baseline glucose, history of diabetes, operation duration, type of surgery and anesthesia method between the peak glucose (determined by RCS) and postoperative composite infections.

All statistical analyses were performed using the statistical software program R version 4.1.1 (The R Foundation for Statistical Computing, https://www.r-project.org/). Statistical significance was defined as 2-sided *P* < 0.05.

## 4. Sample size considerations

No prior statistical power calculation was conducted before the study due to a retrospective study. The sample size was based on the available data. A *post hoc* analysis to assess the effect size we could detect based on actual data was performed. With our current sample size, we had a power of 0.90 or more to detect an odds ratio of infection greater than 1.36 associated with the IH with a type I error of 0.05. Our achieved precision resulted in a narrow CI, ranging from 1.06 to 1.82.

## 5. Results

### 5.1. Patient and surgery characteristics

The final analysis included 3,428 patients (50.41% female) out of 5194 patients identified retrospectively with a median age of 53 years (IQR 46-61; Figure 1). The total number of patients who had at least 1 episode of IH was 536 (15.64%), while 2892 (84.36%) did not. Patient demographics and clinical characteristics categorized by infection or no infection are summarized in Table 1 and Appendix Table B in Supplementary material. The two groups varied substantially in many of the measured variables, with the postoperative infection group generally showing worse health, such as having a higher age, ACCI, EuroSCORE II score, and more comorbidity (Table 1). A higher proportion of IH (30.30% *vs.* 13.14%, *P* < 0.001) and higher peak glucose during surgery (158.27 ± 52.42 mg/dL *vs.* 136.42 ± 40.99 mg/dL, *P* < 0.001) were observed in infection group. It was noted that the infection group had a longer operation and CPB duration (*P* < 0.001). Importantly, an emergency procedure is more likely to develop a postoperative infection (18.91% *vs.* 5.49%, *P* < 0.001). No intraoperative hypoglycemia was observed in both groups. More intraoperative characteristics in patients who did and did not develop infection are summarized in Appendix Table B in Supplementary material.

### 5.2. Association of intraoperative hyperglycemia with postoperative infections

**FIGURE 2:**
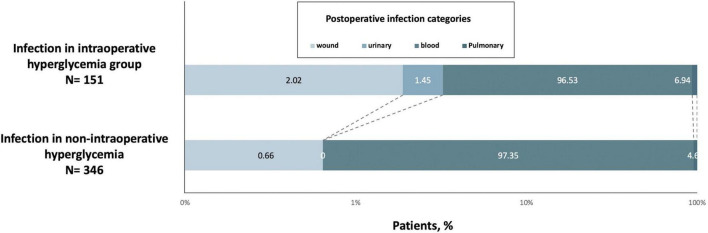
Postoperative infection categories the intraoperative hyperglycemia group (*n* = 151) or control (*n* = 346) groups.

A total of 497 of 3,428 (14.50%) patients developed an infection after cardiac surgery with CPB. Moreover, 151 (28.17% of 536 patients) hyperglycemic patients developed an infection compared with 346 of 2,892 (11.96%) normoglycemic patients (Table 2). Infections included pulmonary (*n* = 497), urinary (*n* = 5), wound (*n* = 8), and blood (*n* = 31), indicating pulmonary infection is the most common complication. The classification of infection complications in all groups is shown in Figure 2.

**TABLE 2:** Risk of postoperative infection with intraoperative hyperglycemia compared with unexposed individuals.

Model information	No. of patients with event/ Total no. of patients		Adjusted OR (95% CI)	*P*-value	
	**Patients with intraoperative hyperglycemia**	**Patients without intraoperative hyperglycemia**			
**Model 1**: Adjusted for demographic factors (age, sex, and BMI)			2.92 (2.33, 3.65)	<0.001	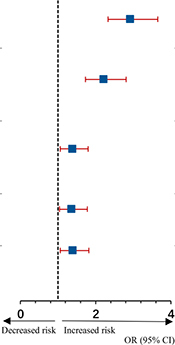
**Model 2**: Model 1+ preoperative comorbidities (i.e., age-adjusted Charlson index, DM, CHD, COPD, etc.)			2.21 (1.73, 2.81)	<0.001	
**Model 3**: Model 2+ surgical factors (i.e., operation type, anesthesia, operation duration, etc.)	151/536 (28.17%)	346/2892 (11.96%)	1.38 (1.06, 1.80)	0.017	
**Model 4**: Model 3+ preoperative laboratory examination			1.36 (1.04, 1.78)	0.025	
**Model 5:** Model 4+ intraoperative blood volume and vasoactive agents			1.39 (1.06, 1.82)	0.016	

Data are n/N (%), unless otherwise specified. ORs (95% CI) were derived from logistic regression models, adjusted for covariates listed in the model information column. BMI, body-mass index; CHD, congenital heart disease; COPD, chronic obstructive pulmonary disease; CI, confidence interval; DM, diabetes mellitus; OR, odds ratio.

**FIGURE 3:**
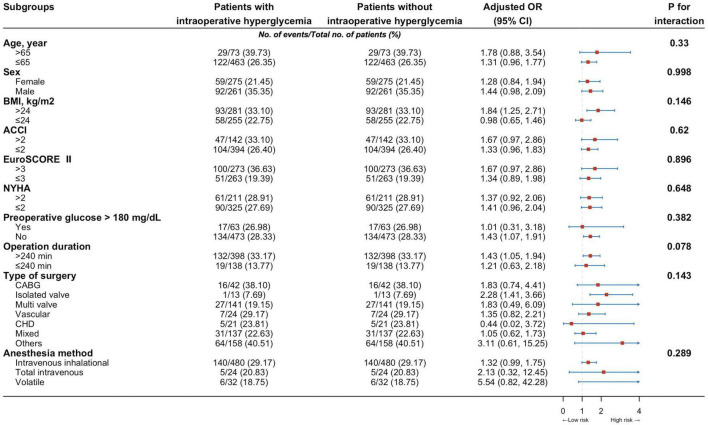
Risk of infection among individuals with intraoperative hyperglycemia by different characteristics, compared with that of individuals without such conditions. ACCI, Aged-adjusted Charlson Index; BMI, body-mass index; CABG, coronary artery bypass grafting; CHD, congenital heart disease; CI, confident interval; EuroSCORE II, European system for cardiac operative risk evaluation II; NYHA, New York Heart Association Classification; OR, odds ratio.

These proportions in the group of people with IH corresponded to an age-adjusted, sex-adjusted, and BMI-adjusted OR of 2.92 (95% CI 2.33-3.65) for composite in-hospital infection cases (model 1, Table 2), which decreased to 2.21 (1.73-2.81) when adding somatic comorbidities into the models (model 2) and then declined to 1.36 (1.04-1.78) after adjusting for more confounders (models 3 and 4). The fully adjusted OR (model 5) was 1.39 (95% CI 1.06-1.82; *P* = 0.016; Table 2). Besides, the elevated risk of infection in individuals with IH did not differ by age group, sex, BMI, ACCI, EuroSCORE II, New York Heart Association (NYHA) class, preoperative glucose, operation duration, type of surgery and anesthesia method (Figure 3).

**FIGURE 4:**
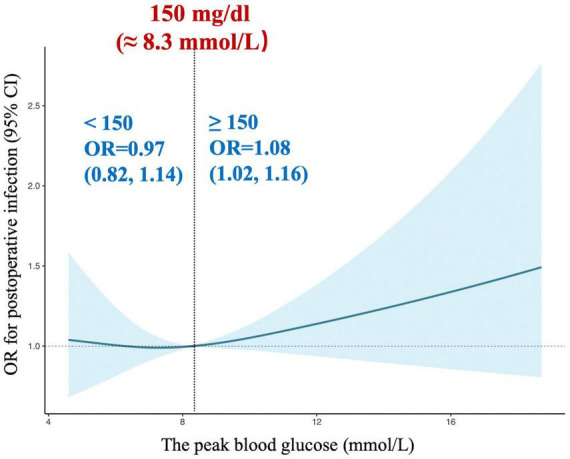
Association of intraoperative peak glucose with postoperative infection. OR, odds ratio.

Next, restricted cubic splines were used to flexibly model and visualize the relation between peak glucose during surgery and composite in-hospital infection (Figure 4). The risk of postoperative composite in-hospital infection was relatively flat around 150 mg/dL of peak glucose during surgery. However, the risk started to increase rapidly above 150 mg/dL (*P* for non-linearity = 0.021). It was observed that above 150 mg/dL, the ORs for composite in-hospital infection per standard deviation of peak glucose was 1.08 (1.02 to 1.16). The elevated risk of composite in-hospital infection in individuals with peak glucose greater than 150 mg/dL did not differ by age group, sex, BMI, ACCI, EuroSCORE II, NYHA class, baseline glucose, history of diabetes, operation duration, type of surgery and anesthesia method (all *P* for interaction > 0.05; Appendix Table C in Supplementary material).

## 6. Discussion

Our findings demonstrated that IH (defined as BGC ≥ 180 mg/dL) is associated with an increased risk of postoperative composite in-hospital infection after cardiac surgery with CPB. In this study, the overall incidence of postoperative composite infection was 14.50%, whereas 11.96% of normoglycemic patients and 28.17% of hyperglycemic patients developed an infection, respectively. Our results suggested that a moderate glycemic control (BGC less than 150 mg/dL) may be ideal for patients undergoing cardiac surgery with CPB.

It has been reported that postoperative infection is considered a major complication after cardiac surgery with CPB. Thus, identifying modifiable risk factors associated with postoperative infection could significantly improve patients’ prognosis. However, the literature is limited and comprise conflicting data on postoperative infection and the associated risk factors in cardiac surgical patients. Moreover, most of the previous studies have been focused on a specific surgery such as on CABG patients, and had limitations in their studies like relatively small sample size and largely inconsistent results, which further restricted their wide applicability (4, 7). The STS guidelines recommend that patients undergoing cardiac surgery with CPB should maintain BGC < 180 mg/dL (11). Notably, IH However, our study showed a J-shaped relation between intraoperative peak glucose and postoperative composite in-hospital infection. The plot revealed a substantial increase in risk within the higher range of intraoperative peak glucose, which lowered around 150 mg/dL and then increased thereafter (*P* for non-linearity = 0.021). Above 150 mg/dL, the ORs per standard deviation for the difference of intraoperative peak glucose was 1.08. Our findings were consistent with previously reported data. For example, a study in critical care that included cardiac surgical patients observed that tight glucose control (80-110 mg/dL) in the perioperative period contributes to worse mortality than standard control (≤ 180 mg/dL) (23). Besides, a review that assessed the effect of tight glucose control (80-110 mg/dL) in surgical patients, including postoperative cardiac surgical patients, concluded that a glucose level ranging from 140 to 180 mg/dL was associated with the best risk-benefit ratio (24). Therefore, these results suggested that tighter glycemic control might be deserved mentioned.

Although there is consensus that tighter glycemic control in cardiac surgical patients improves outcomes, the optimal target for serum glucose levels is unknown. A study involving 4,658 patients underwent isolated CABG, and patients were stratified into three postoperative glycemic groups: tight (≤ 126 mg/dL), moderate (127-179 mg/dL), and liberal control group (≥ 180 mg/dL) (6). This study concluded that moderate glycemic control was superior to a tight or liberal glycemic control group, including decreased mortality and major complications and might be ideal for patients undergoing isolated CABG. These findings were consistent with our current findings, which suggested maintaining glucose levels of less than 150 mg/dL. Similarly, a study evaluated the effect of a tight glucose control strategy (BGC ranged from 90 to 110 mg/dL) in open cardiac surgical patients and revealed that the implementation of glycemic control of 120-180 mg/dL in the perioperative period into clinical practice was not associated with increased morbidity. However, it was associated with a 2-fold increase risk of renal failure or renal failure requiring dialysis (10). These results indicated that stricter glycemia control is not the best solution. However, several studies have attempted to address the dichotomy of strict or less strict glucose control strategy, leading to the evolution of two treatment dogmas. The first treatment strategy included tight glycemic control (upper threshold of 120 mg/dL), while the second comprised normoglycemic or moderate glycemic control (upper threshold of 200 mg/dL) (6, 25, 26). Although these studies are not specific to intraoperative glycose control of the cardiac surgical patient, they do provide some clues. In the current study, we suggested that intraoperative BGC less than 150 mg/dL, a moderate glycemic control target value, may be ideal for patients undergoing cardiac surgery with CPB. Our findings are also consistent with the released American College of Physicians guidelines for intensive insulin therapy in hospitalized patients. However, the guidelines are not specific to the cardiac surgical patient; the BGC management strategy in the perioperative period is addressed. These guidelines suggested that the BGC should be kept between 140 and 200 mg/dL. The average BGC level was 133 ± 17 (range 98-183) in the liberal group during the first 24 h after surgery, 134 ± 20 (range 95-181) for the next 24 h, and 124 ± 18 (range 90-166) for another 24 hours, and our findings are closely in accordance with these recommendations.

The findings in our study raise many questions that should be investigated in the future. The prospective studies should explore the reason behind certain patients developing IH and others not and whether the increased risk of postoperative infection is directly attributable to the presence of IH. Moreover, the studies should examine whether the duration or severity of hyperglycemia affects prognosis. These questions highlighted the need for a larger follow-up prospective study in which the patients are randomized to receive either improved intraoperative glucose control or standard therapy, which is followed by postoperative infection rate determination. In addition, whether the practice of maintaining BGC under 150 mg/dL during cardiac surgery with CPB would reduce the risk of postoperative infection still needs to be validated in prospective, randomized controlled trials.

There are several limitations in this study to be noted. The study was conducted retrospectively, which made it susceptible to inherent biases in patient recruitment and data collection. However, all possible means to minimize any potential bias were taken into consideration: such as enrollment consecutive patients, looked at objective endpoints with predefined rigorous definitions, and double-extracted the data (independently and, for outcomes). In addition, our results should be assessed in a broader array of settings, since on-pump cardiac surgery is a complicated process, and institutions can vary substantially in their strategies of transfusion and CPB, pharmacologic support, and postoperative management. We didn’t differ between deep sternal wound infections and general surgical side infections, because the new onset of wound infections after surgery was determined by medical record review, which was difficult to distinguish the difference between them. But outcome diagnosis was strictly determined according with the definitions and criteria established by the EPCO definitions, and the confounder have been fully considered. The colloid priming for the extracorporeal circulation circuit was 4% succinylated gelatin, which had relatively little effect on levels of glucose without starch. Moreover, as previously discussed more fully in the preceding paragraph, in an observational study, one could only examine associations and cannot attribute causation. Finally, we are sorry for not collecting information on patients with insulin dependent diabetes, non-insulin dependent diabetes due to the nature of the retrospective study.

## 7. Conclusion

In summary, our results showed that a single episode of IH (BGC ≥ 180 mg/dL) was independently associated with postoperative infection after cardiac surgery with CPB. Importantly, we found that BGC less than 150 mg/dL, a moderate glycemic control target value, may be deserved considered as an optimal glucose control value during cardiac surgery with CPB. Our study has relevant findings that provide a potential lever for intervention and behavior change in perioperative. However, further study should be conducted to demonstrate whether the practice of maintaining BGC under 150 mg/dL during cardiac surgery with CPB would reduce the risk of postoperative infection. Such studies could provide insights into clinical guidelines for pain management strategies associated with surgery. Moderated strict glucose control during cardiac surgery with CPB may be better, but the jury is still out.

## Data availability statement

The original contributions presented in this study are included in this article/Supplementary material, further inquiries can be directed to the corresponding author.

## Ethics statement

The studies involving human participants were reviewed and approved by the institutional review boards at West China Hospital of Sichuan University. Written informed consent for participation was not required for this study in accordance with the national legislation and the institutional requirements.

## Author contributions

JS: conceptualization, funding acquisition, and Writing—review and editing. XX, DC, and SC: data curation. DC: formal analysis and methodology. XX, SC, and LQ: investigation. XX and JS: project administration. JS: resources. XX and DC: writing – original draft. All authors contributed to the article and approved the submitted version.
