# Supplementary material for: Association of intraoperative hyperglycemia with postoperative composite infection after cardiac surgery with cardiopulmonary bypass: A retrospective cohort study
Source: Front Cardiovasc Med. 2023 Jan 13;9:1060283. doi: 10.3389/fcvm.2022.1060283 (PMC9880037; doi:10.3389/fcvm.2022.1060283)
Supplement: Supplementary file 1 [file Data_Sheet_1.docx]

**Appendix**

**Appendix Table A. Definition of postoperative complications.**

| **Surgical site infection**: meets the following criteria:  1) Infection occurs within hospital after surgery.  2) The infection appears to be related to the surgical procedure and involves any part of  the body, including the skin incision, facia or muscle layers opened or manipulated.  3) The patient has at least one of the following:   1. Purulent drainage from the surgical site, including the superficial incision, the deep incision, and the organ/space component of the surgical site. 2. Organisms isolated form an aseptically obtained culture of fluid or tissue from the surgical site. 3. At least one of the following symptoms or signs of infection: fever (>38℃), pain or tenderness, localized swelling, redness or heat, or culture-positive finding. 4. Diagnosis of an incisional surgical site infection by a surgeon or attending physician. |
| --- |
| **Pulmonary infection**: meets the following criteria:  1) Sputum culture positive.  2) Respiratory infection. Patient has received antibiotics for a suspected respiratory infection and met one or more of the following criteria: new or changed sputum, new or changed lung opacities, fever, white blood cell count > 12×10^9^ l^-1^. |
| **Bloodstream infection**: laboratory conﬁrmed bloodstream infection as one which meets at least one of the following criteria which should not be related to infection at another site:  1) Patient has a recognized pathogen cultured from one or more blood cultures and the organism cultured from blood is not related to an infection at another site  2) Patient has at least one of the following signs or symptoms: fever >38℃, chills or hypotension, and at least one of the following:   1. Common skin contaminant cultured from two or more blood cultures drawn on separate occasions. 2. Common skin contaminant cultured from at least one blood culture from a patient with an intravascular line, and the physician institutes appropriate antimicrobial therapy. 3. Positive blood antigen test. |
| **Urinary tract infection**: a positive urine culture of ≥10^5^ colony forming units ml^-1^ with no more than two species of micro-organisms, and with at least one of the following symptoms or signs: fever (>38℃), urgency, frequency, dysuria, suprapubic tenderness, costovertebral angle pain or tenderness with no other recognized cause. |

**Appendix Table B: Preoperative Laboratory Examination and Intraoperative Blood Volume and Vasoactive Agents Stratified by Composite Primary Outcome.**

| **Characteristics** | **All**  **N=3428** | **Any infection** | | ***P* value** |
| --- | --- | --- | --- | --- |
|  |  | **Yes (n=497)** | **No (n=2931)** |  |
| **Intraoperative laboratory examination** |  |  |  |  |
| Preoperative HB, g/dL, median [IQR] | 135.00 [122.00; 147.00] | 133.00 [118.00; 146.00] | 135.00 [123.00; 147.00] | 0.018 ^a^ |
| Preoperative NT-pro BNP, pg/ml, median [IQR] | 509.50 [135.00; 1395.25] | 977.00 [329.00; 2409.00] | 451.00 [120.00; 1290.50] | <0.001 ^a^ |
| Preoperative cTnT, ug/L, median [IQR] | 10.70 [7.00;18.20] | 15.60 [10.10; 31.90] | 10.00 [6.60;16.70] | <0.001 ^a^ |
| Preoperative Albumin, mmol/L, median [IQR] | 43.00 [40.30; 45.60] | 41.40 [38.30; 44.30] | 43.20 [40.60; 45.80] | <0.001 ^a^ |
| Preoperative Scr, umol/L, median [IQR] | 76.00 [64.00; 89.00] | 83.00 [69.00; 99.00] | 74.00 [64.00; 87.00] | <0.001 ^a^ |
| **Anesthesia method, n (%)** |  |  |  | 0.002 ^b^ |
| Intravenous inhalational | 2905 (84.74) | 447 (89.94) | 2458 (83.86) |  |
| Total intravenous | 326 (9.51) | 28 (5.63) | 298 (10.18) |  |
| Volatile | 197 (5.75) | 22 (4.43) | 175 (5.96) |  |
| **Intraoperative fluid management** |  |  |  |  |
| Intraoperative RBC infusion, n (%) | 372 (10.85) | 115 (23.14) | 257 (8.77) | <0.001 ^b^ |
| Intraoperative plasma infusion, n (%) | 348 (10.15) | 112 (22.54) | 236 (8.05) | <0.001 ^b^ |
| Intraoperative cryoprecipitate infusion, n (%) | 24 (0.70) | 5 (1.01) | 19 (0.65) | 0.379 ^b^ |
| Intraoperative platelet infusion, n (%) | 1099 (32.06) | 270 (54.32) | 829 (28.28) | <0.001 ^b^ |
| Autotransfusion, mL, median [IQR] | 300.00 [250.00; 500.00] | 400.00 [300.00; 600.00] | 300.00 [250.00; 500.00] | <0.001 ^a^ |
| Crystalloid, mL, median [IQR] | 700.00 [500.00; 1000.00] | 800.00 [500.00; 1100.00] | 700.00 [500.00; 900.00] | <0.001 ^a^ |
| Colloid, mL, median [IQR] | 0.00 [0.00; 200.00] | 0.00 [0.00; 200.00] | 0.00 [0.00; 200.00] | 0.459 ^a^ |
| Urinary, mL, median [IQR] | 800.00 [500.00; 1200.00] | 950.00 [600.00; 1300.00] | 800.00 [500.00; 1200.00] | 0.003 ^a^ |
| **Intraoperative vasoactive agents** |  |  |  |  |
| Epinephrine, n (%) | 2985 (87.08) | 452 (90.95) | 2533 (86.42) | 0.007 ^b^ |
| Norepinephrine, n (%) | 829 (24.18) | 211 (42.46) | 618 (21.06) | <0.001 ^b^ |
| Millinon, n (%) | 396 (11.55) | 112 (22.54) | 284 (9.69) | <0.001 ^b^ |
| Dopamine, n (%) | 19 (0.55) | 5 (1.01) | 14 (0.48) | 0.180 ^b^ |
| Nitroglycerin, n (%) | 2349 (68.52) | 306 (61.57) | 2043 (69.70) | <0.001 ^b^ |

Data are present in n (%) or median [IQR].

P values are derived from: a. u-test, ^b^ chi-square test or Fisher exact test.

Abbreviation: cTnT, Cardiac troponin T; HB, hemoglobin; IQR, interquartile range; NT-pro BNP, N-terminal pro-brain natriuretic peptide; RBC, red blood cell; Scr, serum creatinine concentration.


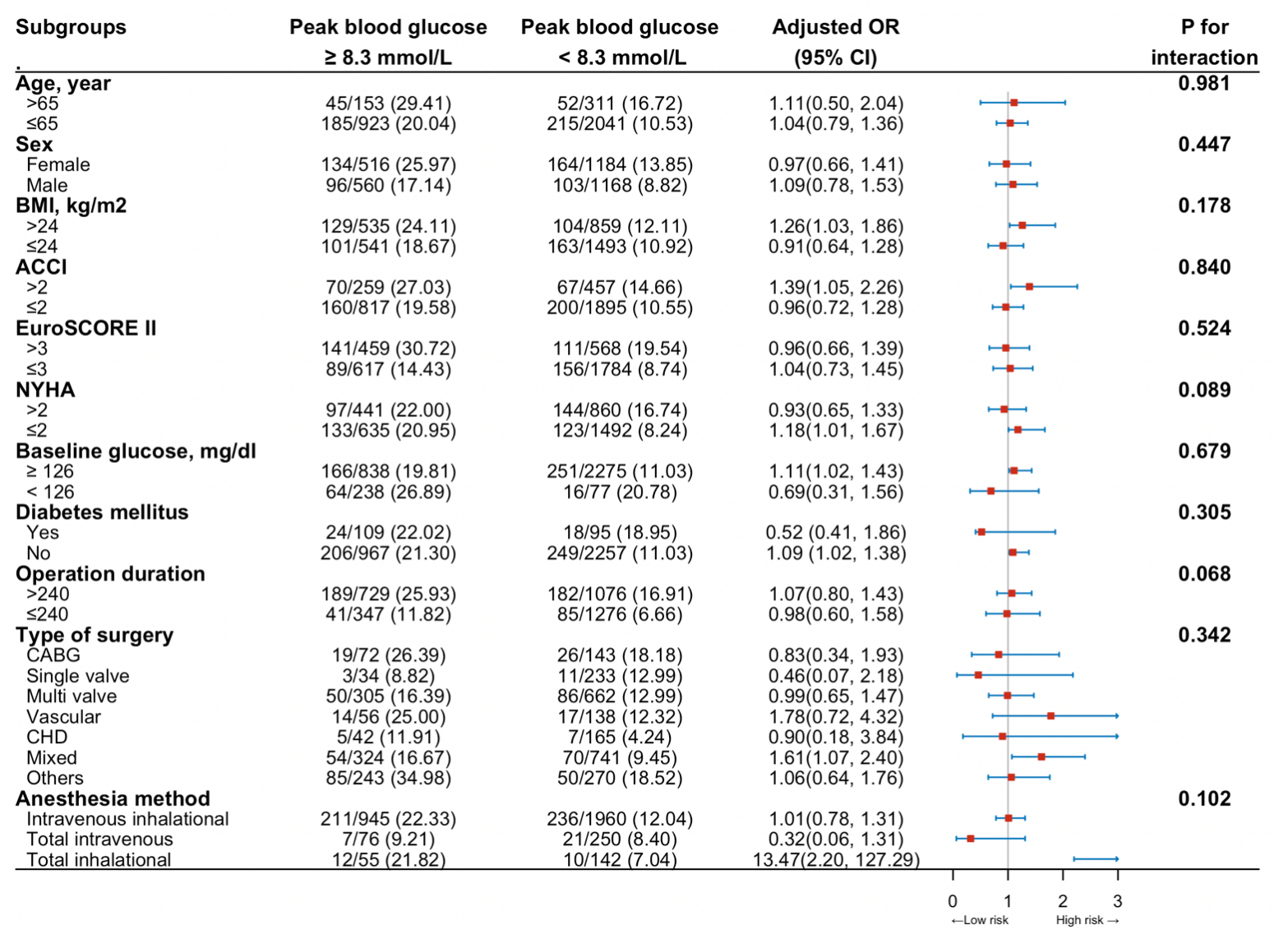
**Appendix Table C. Risk of postoperative infection among individuals with peak blood glucose ≥ 8.3 mmol/L by different baseline glucose and history of diabetes, compared with that of individuals without such conditions.**

Abbreviations: ACCI, Aged-adjusted Charlson Index; BMI, body-mass index; CABG, coronary artery bypass grafting; CHD, congenital heart disease; CI, confident interval; EuroSCORE II, European system for cardiac operative risk evaluation II; NYHA, New York Heart Association Classification; OR, odds ratio.
